# Radiographic Severity and Clinical Implications of Knee Osteoarthritis in Adults Under 55: Incidence and Insight Into Potential Candidates for Geniculate Artery Embolisation

**DOI:** 10.7759/cureus.95638

**Published:** 2025-10-28

**Authors:** David J Solomon, Peter McLoughlin

**Affiliations:** 1 Trauma and Orthopaedics, Ulster Hospital Dundonald, Belfast, GBR; 2 Radiology, Northern Ireland Medical and Dental Training Agency, Belfast, GBR

**Keywords:** arthroplasty, geniculate artery embolisation, knee osteoarthritis, oa, osteoarthritis, young oa

## Abstract

Background: Knee osteoarthritis (OA) increasingly affects adults well before traditional "older-age" brackets, raising questions about joint-preserving options. We evaluated radiographic OA severity among patients <55 years and considered the potential role of geniculate artery embolisation (GAE) for symptomatic management.

Methods: This was a retrospective cross-sectional study of consecutive outpatients (<55 years) referred to a Northern Ireland hospital with suspected knee OA. Anteroposterior radiographs were graded according to the Kellgren-Lawrence (KL) scale. Outcomes were the proportion with radiographic OA (KL≥2) and with moderate-to-severe OA (KL≥3).

Results: A total of 89 patients (mean age 47.5 years; range 35-54) were included in the study. The distribution of KL grades was as follows: grade 0 in 40.4%, grade 1 in 5.6%, grade 2 in 20.2%, grade 3 in 29.2%, and grade 4 in 4.5% of patients. Overall, 53.9% had radiographic osteoarthritis (KL ≥2), while 33.7% had moderate-to-severe disease (KL ≥3).

Conclusion: Over half of symptomatic outpatients under 55 had definite radiographic OA, and one-third had moderate-to-severe disease, highlighting a substantial disease burden in "younger" adults. Given joint-replacement deferral in this age group, GAE warrants evaluation within research pathways for persistent, treatment-refractory pain. The National Institute for Health and Care Excellence (NICE) currently recommends GAE only in research settings.

## Introduction

Knee osteoarthritis (OA) is among the leading causes of pain and disability worldwide, and its burden is rising [1]. Although prevalence increases with age, multiple cohorts now document substantial disease in middle-aged adults (often beginning in the 30s-50s), driven by injury, obesity, and occupational/sport exposures. Recent global estimates indicate a high incidence and prevalence of knee OA, even among adults aged 30-44 [2,3].

Population studies in the US and UK report notable radiographic knee OA in those ≥45, with a cumulative five-year incidence of around 18% in 45-64-year-old women in the Chingford study [3]. However, less is reported on radiographic severity profiles among symptomatic patients younger than those typically considered for arthroplasty, especially in UK secondary care.

As joint replacement is often deferred in <55-year-olds, there is considerable interest in joint-preserving interventions. Geniculate artery embolisation (GAE) targets synovial neovascularity and inflammatory hyperaemia. Early studies, including a sham-controlled trial, suggest improvements in pain and function [4], but guidance in the UK currently limits use to research settings pending more robust comparative data [5].

The primary objective of this study was to describe the radiographic burden and severity of knee OA among adults <55 years presenting with knee pain in a secondary care setting in Northern Ireland (NI). The secondary objective was to explore the potential relevance of these findings for GAE candidacy, given the emerging evidence supporting its role in symptom management for moderate osteoarthritis. To ensure the reproducibility and reliability of radiographic grading, all images were independently reviewed by radiology consultants or senior registrars, with consensus on Kellgren-Lawrence (KL) scores.

## Materials and methods

This retrospective cross-sectional study was conducted over a one-month period (July 2025), during which all consecutive outpatient referrals to the radiology department of Belfast City Hospital, Northern Ireland, for suspected knee OA were included. Participants included adults aged <55 years at presentation with knee radiographs available for review. A consecutive sampling approach was used to minimise selection bias.

Inclusion and exclusion criteria

The inclusion criteria comprised all patients younger than 55 years who were referred for plain radiographs due to suspected OA in July 2025. Exclusion criteria were radiographs obtained for acute trauma, suspected or confirmed fracture, known inflammatory arthropathy (e.g., rheumatoid arthritis, psoriatic arthritis), prior knee arthroplasty, or inadequate radiograph quality precluding reliable grading. Patients with prior knee surgical hardware and those over 55 years old at the time of radiograph were also excluded.

Imaging protocol

The imaging protocol included standard anteroposterior (AP) weight-bearing knee radiographs, which were independently assessed by radiology consultants and senior registrar assessors trained in the application of the KL grading system. Radiographs were graded by the assessors using the KL system (0-4). Any discrepancies were resolved by consensus discussion.

Radiographic OA was defined as a KL grade of 2 or higher, while moderate-to-severe disease was defined as a grade of 3 or higher. Representative radiographs of grades 3 and 4 OA are depicted in the Results section.

Statistical analysis

The primary outcomes were the proportions of patients with radiographic OA (KL grade ≥2) and with moderate-to-severe OA (KL grade ≥3). For these outcomes, point estimates were calculated alongside 95% confidence intervals using the Wilson binomial method. Analysis was conducted using standard statistical software.

## Results

A total of 89 patients under the age of 55 were included in the analysis, with a mean age of 47.5 years (range 35-54). The cohort consisted of 54 females (60.7%) and 35 males (39.3%). The largest subgroup was aged 50-55 years (41/89; 46.1%), followed by those aged 45-50 years (27/89; 30.3%) (Table 1). When stratified by age, the prevalence of radiographic osteoarthritis (KL grade ≥2) increased progressively from the 35-40 year group to the 50-55 year group. Most moderate-to-severe (KL grade 3-4) cases occurred in patients aged 45 years or older. Although females represented the majority of the cohort (60.7%), no marked sex-related difference in radiographic severity was observed. However, a slightly higher proportion of KL grade 3-4 was noted among older female patients.

**Table 1:** Age and Sex Distribution of Patients Under 55 With Suspected Knee Osteoarthritis (OA) Values are presented as absolute numbers (n). The table demonstrates the distribution of patients stratified by five-year age groups and sex. The largest subgroup was aged 50-55 years (41/89, 46.1%), followed by those aged 45-50 years (27/89, 30.3%). Overall, 54 (60.7%) of the cohort were female and 35 (39.3%) were male.

Age Group (Years)	Female (n)	Male (n)	Total (n)
30–35	0	0	0
35–40	5	5	10
40–45	6	5	11
45–50	19	8	27
50–55	24	17	41
Total	54	35	89

**Figure 1:**
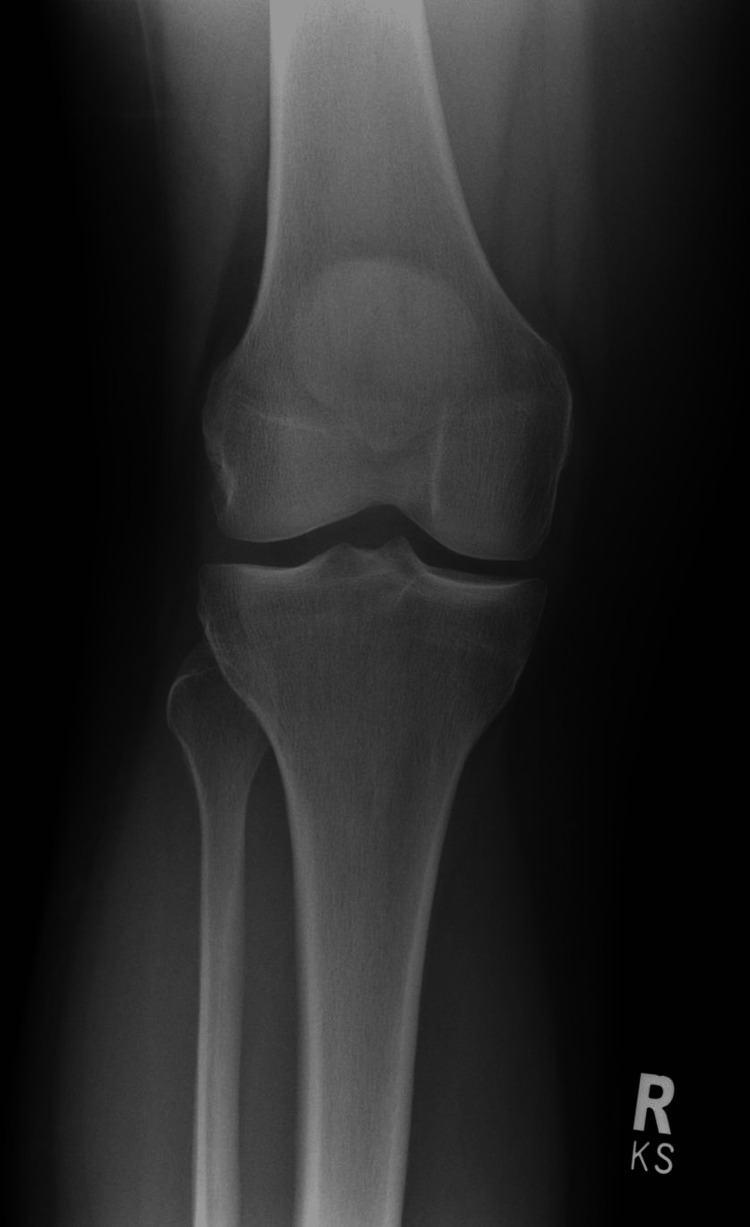
Representative radiograph of Kellgren-Lawrence grade 0 knee osteoarthritis Anteroposterior weight-bearing radiograph showing a normal joint with preserved joint spaces, smooth articular margins, and no osteophyte formation or subchondral sclerosis. This appearance is consistent with Kellgren-Lawrence grade 0, indicating the absence of radiographic osteoarthritis.

**Figure 2:**
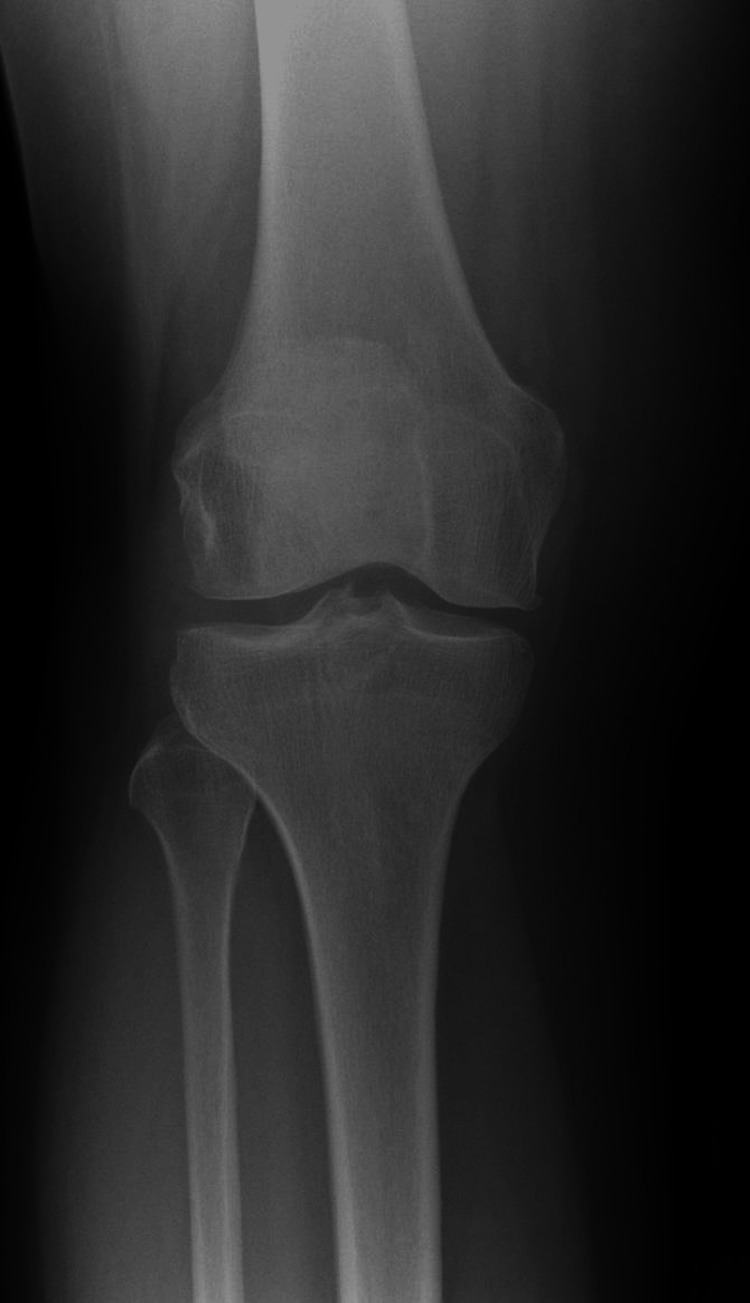
Representative radiograph of Kellgren-Lawrence grade 1 knee osteoarthritis Anteroposterior weight-bearing radiograph demonstrating minute osteophyte formation at the tibial margins and some joint-space narrowing. Findings correspond to Kellgren-Lawrence grade 1, representing early osteoarthritic change.

**Figure 3:**
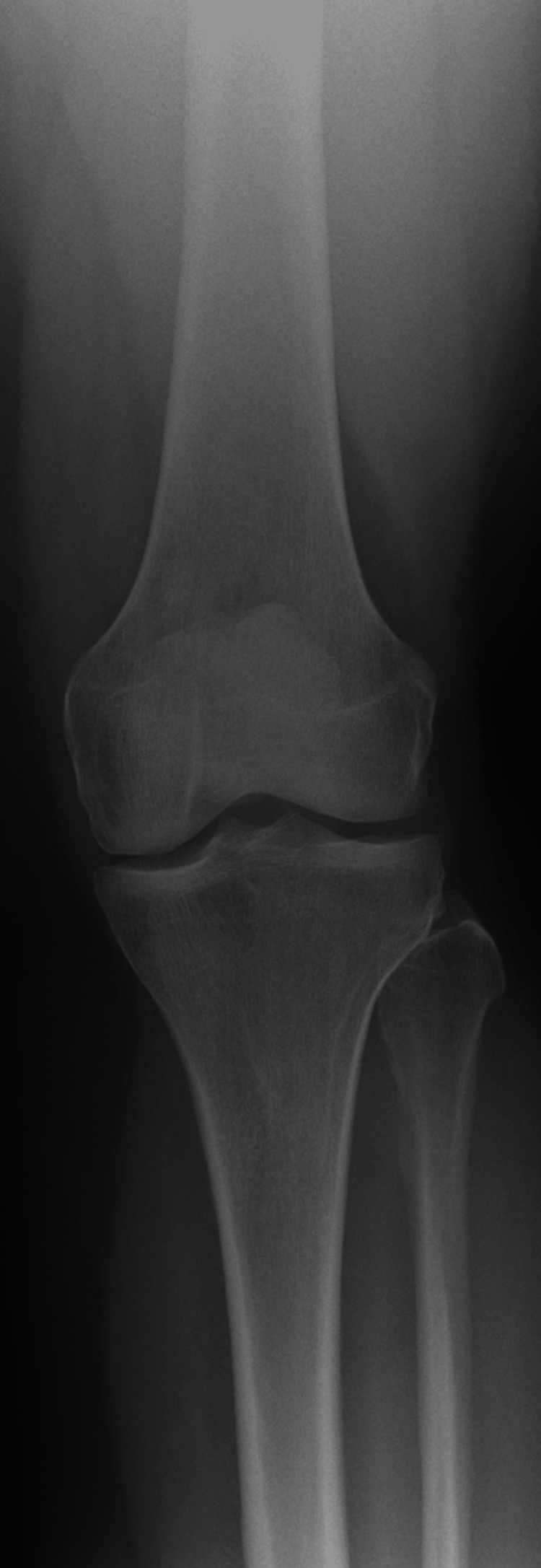
Representative radiograph of Kellgren-Lawrence grade 2 knee osteoarthritis Anteroposterior weight-bearing radiograph showing some osteophyte formation with joint-space narrowing and early subchondral sclerosis. These features meet the criteria for Kellgren-Lawrence grade 2 mild osteoarthritis.

**Figure 4:**
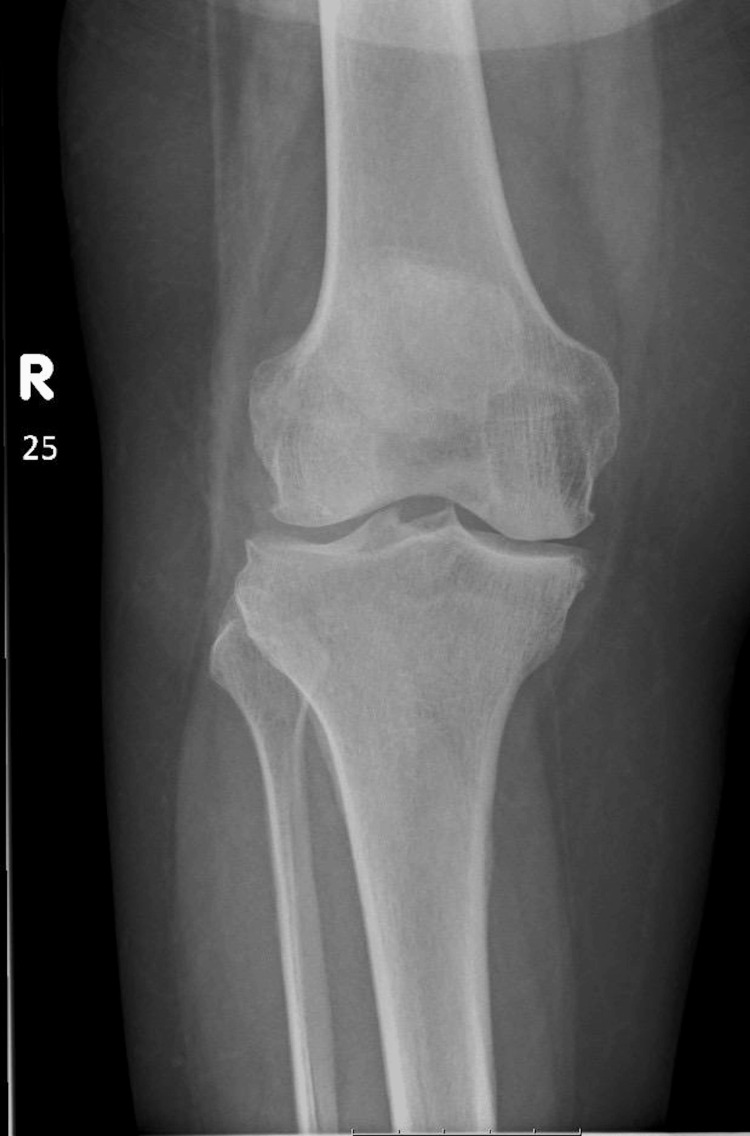
Representative radiograph of Kellgren-Lawrence grade 3 knee osteoarthritis Anteroposterior weight-bearing knee radiograph demonstrates moderate osteoarthritis with definite joint space narrowing, multiple osteophytes, and early subchondral sclerosis. Findings are consistent with Kellgren-Lawrence grade 3 changes.

**Figure 5:**
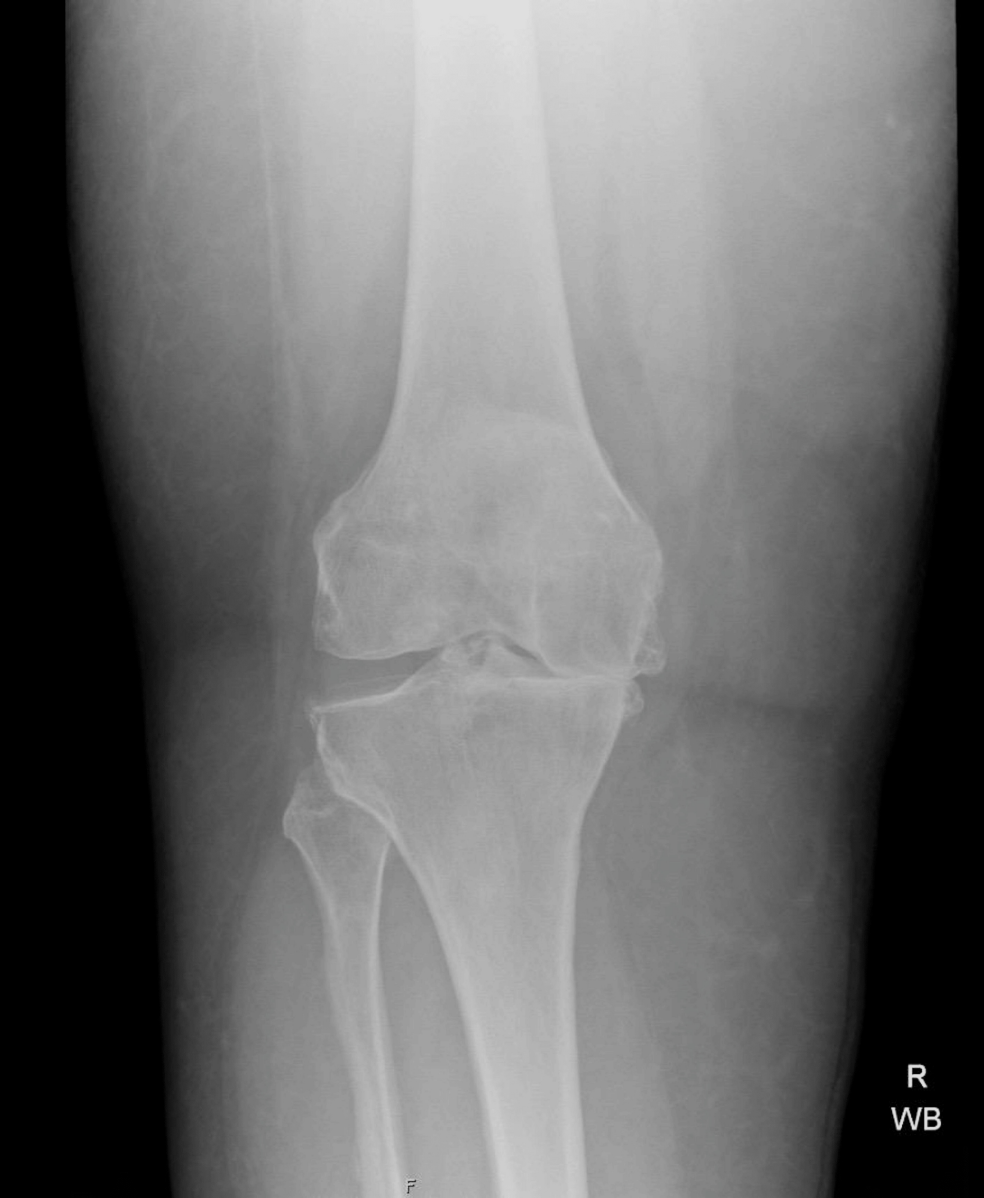
Representative radiograph of Kellgren-Lawrence grade 4 knee osteoarthritis Anteroposterior weight-bearing knee radiograph demonstrates severe osteoarthritis with marked joint space loss, large osteophyte formation, and pronounced subchondral sclerosis. Findings are consistent with Kellgren-Lawrence grade 4 changes.

Radiographic severity was assessed using the KL system (Figures 1-5). The distribution was as follows: KL 0 in 36 patients (40.4%), KL 1 in five patients (5.6%), KL 2 in 18 patients (20.2%), KL 3 in 26 patients (29.2%), and KL 4 in four patients (4.5%) (Table 2). When grouped by diagnostic threshold, 53.9% of the overall cohort (48/89; 95% CI 43.6-63.9) demonstrated radiographic OA (KL ≥2). Within this group, 33.7% of patients (30/89; 95% CI 24.7-44.0) had moderate-to-severe OA (KL ≥3). The prevalence of radiographic OA increased with age, with the majority of KL 3 and KL 4 cases observed in patients aged 45 years or older.

**Table 2:** Distribution of Kellgren-Lawrence Grades in Patients Under 55 Years With Suspected Knee Osteoarthritis (OA) Kellgren-Lawrence grade osteoarthritis in < 55-year-olds.

Kellgren-Lawrence Grade	Number of Patients (n)	Percentage (%)
0	36	40.4
1	5	5.6
2	18	20.2
3	26	29.2
4	4	4.5

These findings confirm that more than half of symptomatic outpatients under the age of 55 presenting with knee pain already have radiographic evidence of OA, and a substantial proportion demonstrate moderate-to-severe disease that may influence clinical decision-making and candidacy for joint-preserving interventions.

## Discussion

In symptomatic outpatients under 55, more than one in two demonstrated definite radiographic OA, and one in three had moderate-to-severe changes. This underscores a high burden of structurally evident disease in a group where arthroplasty is often deferred due to concerns regarding long-term implant durability and increased risk of revision. The rising incidence of knee OA in younger adults reflects both lifestyle and injury-related risk factors, highlighting the need for effective joint-preserving treatment options [6].

Conservative management remains the cornerstone of care for this population, including structured exercise therapy, weight management, activity modification, and pharmacological or injection-based analgesia. However, when these measures fail, clinicians and patients face limited alternatives before resorting to arthroplasty. In this context, GAE involves selective catheterisation of the genicular arterial branches supplying the inflamed synovium, followed by targeted delivery of calibrated microspheres or particles to reduce synovial hypervascularity and inflammatory mediators. The procedure is typically performed via femoral access under fluoroscopic guidance, using cone-beam CT or angiography to confirm appropriate vessel occlusion, therefore reducing a key pathological driver of inflammation and pain in OA. Careful angiographic evaluation is essential to avoid non-target embolisation of the skin or osseous branches supplying the subchondral bone, as these may theoretically predispose to osteonecrosis. Published trials and systematic reviews report a low incidence of complications, with no confirmed cases of osteonecrosis to date when appropriate microcatheter technique and particle size selection are used [7-12].

Evidence supporting GAE has expanded considerably in recent years. The randomised, triple-blind controlled trial by Landers et al. (2023) demonstrated clinically meaningful improvements in pain and function among patients with early-stage OA [7]. More recently, Cusumano et al. (2024) reported sustained improvements in pain and functional scores over two years in a prospective trial, with acceptable safety outcomes [8]. Systematic reviews and meta-analyses published in 2023 by Taslakian et al. and Epelboym et al. further support GAE as an effective intervention, with statistically significant reductions in pain scores, improved quality of life, and a low incidence of complications [9,10]. A 2025 meta-analysis by Abussa et al. reinforced these findings, though it also highlighted heterogeneity across studies and emphasised the need for larger, sham-controlled RCTs [11].

Patient selection remains critical. Current evidence suggests that individuals with persistent pain despite optimal conservative therapy, radiographic evidence of mild-to-moderate OA (KL 2-3), and imaging signs of synovitis are the most likely to benefit [4]. Inclusion criteria across published studies commonly require pain of at least three to six months' duration and exclusion of those with inflammatory arthropathies or advanced structural collapse. Some trials also stratify patients based on MRI findings, as synovial hypervascularity is a predictor of treatment response. Notably, the GENESIS trial evaluating permanent embolic microspheres reported clinically significant improvements in KOOS and WOMAC scores, sustained for up to 24 months, providing further reassurance on the durability of GAE outcomes [12].

Safety data remain reassuring. Reported complications are generally minor and transient, including skin discolouration, localised swelling, or transient paraesthesia. Rare cases of non-target embolisation and imaging findings suggestive of osteonecrosis have been described, though these often resolve without long-term sequelae. Importantly, no high rates of serious adverse outcomes have been reported in medium-term follow-up studies. Nevertheless, the lack of very long-term safety data, particularly regarding potential impacts on future arthroplasty, remains a limitation [13].

Our findings highlight a substantial cohort of younger, symptomatic patients with moderate radiographic disease who may struggle with persistent pain despite conservative measures. In our cohort, approximately one-third of patients had KL 3 changes and a further 20% had KL 2 changes, representing the radiographic severity group most frequently considered in published GAE trials. In the GENESIS and IDE prospective trials, patients with KL 2-3 disease demonstrated meaningful improvements in WOMAC and KOOS pain and function scores after embolisation, with benefits sustained to 24 months [7,8,12]. Thus, a substantial proportion of our patients would theoretically overlap with the populations shown to benefit from GAE, provided symptoms persisted despite conservative measures. It is noteworthy that a small subset of patients displayed radiographic malalignment, particularly among those with KL grade 4 changes. While such deformity is uncommon in individuals <55 years, it may reflect long-standing mechanical imbalance or undocumented post-traumatic factors.

While no patients in our series underwent embolisation, these correlations highlight a potential candidate pool in secondary care within Northern Ireland, supporting the relevance of exploring GAE within UK research frameworks. In such cases, GAE could represent a useful bridge therapy, potentially delaying the need for arthroplasty while improving quality of life, work capacity, and functional independence. Integration of GAE into practice would require a multidisciplinary framework involving orthopaedics, radiology, and primary care teams to ensure appropriate patient selection and follow-up. Importantly, the National Institute for Health and Care Excellence (NICE) currently recommends that GAE be restricted to research settings [5], underscoring the need for UK-based, high-quality trials to evaluate cost-effectiveness, comparative outcomes, and long-term safety.

Limitations

Retrospective design and single-centre setting limit generalisability. It is important to note that our cohort did not undergo GAE; therefore, no direct clinical outcomes from embolisation can be reported. Additionally, the absence of validated clinical symptom scores such as WOMAC or KOOS, the lack of BMI and comorbidity data, and prior injury history.

Future research should aim to integrate radiographic severity with longitudinal clinical outcomes, BMI, and imaging biomarkers of synovitis to refine the selection criteria for GAE and better characterise outcomes in this younger cohort.

## Conclusions

Among symptomatic outpatients aged under 55, radiographic knee OA is common, and a significant proportion already demonstrate moderate-to-severe disease. This highlights an under-recognised disease burden in a younger group that is often not considered for arthroplasty, reinforcing the need for effective joint-preserving strategies.

GAE has shown promising results in multiple trials and systematic reviews, with sustained benefits for pain and function and a favourable safety profile. For carefully selected, treatment-refractory patients, GAE may serve as a valuable bridge therapy to delay joint replacement. However, widespread adoption should await further high-quality evidence and updated NICE guidance. Multidisciplinary collaboration will be crucial to integrating GAE into clinical pathways and optimising outcomes for younger adults living with symptomatic OA.
